# Effects of Sea Buckthorn Polysaccharides on Rumen In Vitro Fermentation Characteristics and Microbial Composition of Hu Sheep

**DOI:** 10.3390/microorganisms13112639

**Published:** 2025-11-20

**Authors:** Junlin Lan, Zhenzi Xu, Jiahao Li, Xin Li, Yuanyuan Li, Wenju Zhang

**Affiliations:** 1 College of Animal Science and Technology, Shihezi University, Shihezi 832003, China; lanjunlin1232025@163.com (J.L.); 19882955630@163.com (Z.X.); xjauljh@163.com (J.L.); 2 College of Life Sciences, Shihezi University, Shihezi 832003, China; lxin@shzu.edu.cn

**Keywords:** sea buckthorn polysaccharide, in vitro rumen fermentation, fermentation parameters, rumen microbial community

## Abstract

This study evaluated the effects of sea buckthorn polysaccharides (SBP) on rumen fermentation in Hu sheep through in vitro fermentation. A single-factor randomized design was employed with four treatment groups in which SBP was added to a basal diet at 0, 1%, 2% and 3% of the total ration dry matter (DM). Based on gas production, degradation rates, and fermentation parameters, the control group and the 2% group were selected for 16S rDNA sequencing. The results showed that compared with the control group, the 2% SBP treatment significantly increased gas production rate (*p* < 0.05). Addition of 2% and 3% SBP significantly increased DM degradability (*p* < 0.05). SBP supplementation significantly increased fermentation fluid pH (*p* < 0.05) and altered volatile fatty acid profiles, increasing the acetate/propionate ratio as well as the concentrations of butyrate, isobutyrate and valerate. Compared with the control group, the addition of 2% SBP led to significant changes in the microbial composition of the in vitro rumen fermentation fluid. It significantly increased beneficial bacterial phyla and genera, such as Bacteroidetes, *Rikenellaceae_RC9_gut_group* and others, while reducing pathogenic bacteria (*p* < 0.05). Additionally, functional prediction revealed that the SBP group was significantly enriched in pathways related to carbohydrate metabolism, biosynthesis of amino acid, and biosynthesis of secondary metabolites (*p* < 0.05). In summary, adding 2% SBP to Hu sheep feed can improve the Rumen fermentation and microbial communities. However, higher doses did not yield additional benefits in these measured indicators.

## 1. Introduction

Rumen fermentation is a central process in the digestive system of ruminants, and its efficiency directly influences the conversion rate of nutrients and animal production performance [1]. The ruminal microbiota play an important role in the digestion and metabolism of ruminants, and its composition and function directly affect feed utilization and host health [2]. With the growing demand for mutton consumption, the sheep farming industry has been steadily developing. During lamb fattening, protein levels in the diet are typically increased to promote growth. However, prolonged high-protein diets may lead to rumen dysfunction and metabolic abnormalities. Therefore, regulating the rumen microbial community to enhance feed nutrient utilization and improve productivity while maintaining a stable and healthy rumen environment has become a current research focus. In recent years, regulating the rumen microbiota to improve nutrient use efficiency, production performance and metabolic function has become a research hotspot. Among them, plant active ingredients have attracted much attention due to their natural, safe, and potential to regulate microbial activity and fermentation characteristics [3]. For example, supplementing Huangqin flavonoids extract in the diet of lactating cows can improve lactation performance and milk quality by improving the internal environment of the gastrointestinal tract and host health [4]. Similarly, inclusion of Jerusalem artichoke polysaccharides in lamb diets can improve the rumen microbial community, promote proliferation of propionate-producing bacteria and suppress methanogen activity, thereby improving nitrogen metabolism efficiency, enhancing muscle protein deposition and favoring accumulation of beneficial fatty acids [5].

Sea buckthorn is native to the Himalayan region and is now distributed across Eurasia. It has a long history of use as a medicinal and edible plant. Rich in polysaccharides, flavonoids and vitamins [6], sea buckthorn has been shown to possess anti-inflammatory [7], antioxidant [8] and immunomodulatory activities [9]. SBP is an important bioactive constituent of the plant. Studies report that SBP can protect against acetaminophen-induced hepatotoxicity via modulation of the *Nrf-2-HO-1-SOD2* signaling pathway [10]. A synbiotic composed of SBP, *Bifidobacterium longum* and *Lactobacillus acidophilus* has been reported to reshape the intestinal microbiota and regulate the Th17/Treg balance, thereby ameliorating DSS-induced colitis [11]. However, research on SBP in ruminants is limited, and its specific mechanisms of action in the rumen, particularly their regulatory effects on microbial community structure and composition, as well as fermentation products, are not yet clear.

Therefore, this study aimed to systematically evaluate the effects of SBP supplementation to the diet on rumen fermentation parameters in Hu sheep, including pH, NH_3_-N, and volatile fatty acid (VFA) production, to clarify the influence of SBP on ruminal fermentation efficiency. Additionally, by exploring the effects of SBP on the composition and structure of microbial communities in sheep rumen fermentation in vitro, the potential pathway of improving feed utilization efficiency by regulating key microbial communities was revealed. This study hypothesizes that dietary SBP supplementation will modulate rumen microbial community structure and metabolic potential, thereby enhancing ruminal fermentation efficiency and feed utilization in Hu sheep. The research results will provide a theoretical basis for the development of green ruminant feed additives and a reference for the high-value utilization of SBP resources.

## 2. Materials and Methods

### 2.1. Materials and Experimental Design

The substrate for rumen fermentation in vitro is a total mixed ration (TMR) for sheep.

Formulate basal diets for each group based on the Nutritional Requirements for Chinese Sheep (NY/T 816-2021) [12]. The composition and chemical composition of the basal diet are shown in Table 1. The basal diet (total mixed ration, TMR) was dried to constant weight at 65 °C in an electric hot-air drying oven (DHG-9420A, Shanghai Yiheng Scientific Instrument Co., Ltd., Shanghai, China). It was then pulverized using a high-speed universal pulverizer (CH-250A, Zhejiang Yongkang Chenhe Shengfeng Industry & Trade Co., Ltd., Yongkang, China) and sieved through a 40-mesh screen to serve as the substrate for subsequent in vitro rumen fermentation. Chemical composition analysis of the diet was conducted according to AOAC [13] methods for DM, nitrogen, and EE, while NDF and ADF were determined using the method proposed by Van Soest et al. [14]. Protein content was calculated by multiplying the nitrogen content by 6.25. DE can be calculated according to NY/T 816-2021 and DE is calculated based on the DE values of each raw material specified in this standard [12]. The experiment was divided into four groups, a control group (fed basal diet without SBP) and three treatment groups (fed basal diet supplemented with 1.0%, 2.0%, or 3.0% SBP, respectively). SBP (≥95% purity) were commercially acquired from Shaanxi Yunqi Biotechnology Co., Ltd., (Xi’an, China), with six replicates per group.

### 2.2. In Vitro Fermentation

This experiment was conducted at the College of Animal Science and Technology, Shihezi University. The experimental protocol and sample collection procedures were approved by the Experimental Animal Ethics Committee of Shihezi University (Approval No.: A2024-726).

Three healthy 3-month-old Hu sheep with similar body weights (24.63 ± 2.28 kg) were selected as rumen fluid donors for the experiment. The donor sheep had free access to feed and water daily. Rumen fluid was collected 2 h before the morning feeding using an oral rumen fluid sampler (Chengdu Huazhi Kaiwu Technology Co., Ltd., Chengdu, China). After discarding the initial saliva-contaminated fraction, the collection bottle was rinsed with a small amount of raw rumen fluid. Fresh rumen fluid was then collected, filtered through four layers of gauze, and transferred into a thermos bottle preheated to 39 °C. To preserve anaerobic conditions, CO_2_ was infused into the flask, which was immediately sealed tightly before being transported to the laboratory. Subsequently, rumen filtrate and artificial buffer were mixed in a volume ratio of 1:2 to prepare rumen inoculum. The preparation was carried out at a constant temperature of 39 °C and continuous introduction of CO_2_ to maintain an anaerobic environment. The artificial rumen buffer solution was prepared in accordance with the method described by Menke et al. [15], this involved the combination of distilled water, microminerals, macrominerals, and a buffer solution. During the experiment, accurately weigh 0.5 g of fermentation substrate on a DM basis and transfer it into a 100 mL glass syringe (HFT000025, Häberle, Schwerte, Germany). The glass syringe was preheated in an electric heating constant temperature drying oven (DHG-9247A, Shanghai Jinghong Laboratory Equipment Co., Ltd., Shanghai, China) at 39 °C. Subsequently, about 30 mL of CO_2_-saturated artificial rumen culture solution was added to the glass syringe. After expelling air bubbles from the syringe, it was sealed, and the initial volume scale was recorded. Fermentation was conducted for 48 h in a water bath shaker (Wat 50; Shanghai Hetaian Instrument Co., Ltd., Shanghai, China) at a temperature of 39 °C and a rotation speed of 45 r/min.

### 2.3. Sample Collection and Analysis

The scale of the syringe piston was recorded at different fermentation time points (2, 4, 6, 8, 10, 12, 24, 36, and 48 h). Meanwhile, according to the method proposed by Mauricio et al. [16], the gas production at each time point was calculated using the following formula:

GP_t_ = 100 × (V_1_ − V_0_)/W_0_,

In the formula, GP_t_ represents the cumulative gas production at time t (mL/g); V_1_ denotes the scale at the syringe plunger at time t (mL); V_0_ is the scale at the syringe plunger of the blank group (mL); W_0_ stands for the DM weight of the sample (g).

The gas production values at different time points were substituted into the Gompertz model equation [17] to fit the gas production kinetic parameters. The equation is:

$${GP}_{t}=Aexp\{-exp[1+\frac{be}{A}(Lag-t)]\}$$

GP_t_ represents the cumulative gas production at time point t (mL); A is the theoretical maximum gas production (mL); b is the gas production rate constant (mL/h); e is the base of the natural logarithm function (2.71828); Lag is the lag time of in vitro fermentation gas production (h); t is the gas production measurement time point (h).

After the fermentation period, the pH of the in vitro fermentation broth was measured using a calibrated portable pH meter (PHSJ-3F, Leici, Shanghai, China). Each sample was measured three times, and the average value was calculated. The NH_3_-N concentration in the fermentation broth was determined using the phenol-hypochlorite colorimetric method [18]. The volatile fatty acid (VFA) content in the fermentation broth was analyzed by gas chromatography (model 6890N, Agilent, Santa Clara, CA, USA). Subsequently, the fermentation residues collected in glass syringes were used to determine the DM and CP contents [13], based on which their respective degradability were calculated. The calculation formula was as follows:

Nutrient degradability (%) = 100 × (Nutrient content before fermentation − Nutrient content after fermentation)/Nutrient content before fermentation.

### 2.4. Amplification, Sequencing, and Analysis of Rumen Bacterial 16S Rdna

After fermentation, collect the culture medium from the glass syringe and transfer it to a 5 mL sterile tube. There are 4 groups in total, with 6 samples in each group, and they are stored in a −80 °C freezer for testing. The in vitro culture samples were sent to Shanghai Meiji Biomedical Technology Co., Ltd. (Shanghai, China) for high-throughput sequencing. The main steps involved the extraction of total genomic DNA from the microbial communities, performed according to the manufacturer’s instructions for the Z.N.A.^®^ soil DNA kit (Omega Bio-tek, Norcross, GA, USA). The quality of the extracted genomic DNA was assessed by 1% agarose gel electrophoresis, while its concentration and purity were determined using a spectrophotometer. PCR amplification of the V3–V4 variable region of the 16S rRNA gene using 338F/806R primers with extracted DNA as a template [19]. Genomic sequencing was performed using the Illumina Miseq PE300 platform (Illumina, Inc., San Diego, CA, USA). The reads obtained from sequencing were spliced according to the overlap relationship, and the sequence quality was controlled and filtered at the same time, then divided into operational taxonomic units (OTUs) according to the similarity of more than 97%. Based on OTUs, the Alpha diversity indices Ace, Chao, Shannon, and Simpson of intestinal microbiota bacteria were analyzed. Principal coordinate analysis (PCoA) was performed using the Bray–Curtis distance algorithm, and Kruskal–Wallis rank sum test was used to analyze the species with significant differences between the two groups.

### 2.5. Data Statistics and Analysis

The experimental data were preliminarily organized using Excel 2021, and one-way analysis of variance was performed using SPSS 26.0 software, with multiple comparisons using Duncan’s method. Polynomial contrast test was used to evaluate the linear and quadratic effects of polysaccharide addition on each index. The significance level was set to *p* ≤ 0.05, and the trend difference was set at 0.05 < *p* ≤ 0.10.

## 3. Results

### 3.1. Effects of SBP on Rumen In Vitro Fermentation Gas Production and Nutrient Degradability of Hu Sheep

The effect of SBP on the in vitro rumen fermentation gas production of sheep over time is shown in Table 2. In all groups, the amount of gas accumulation gradually increased over time, initially showing a rapid increase phase, and then gradually slowing down. The addition of SBP significantly increased the gas generation during the early fermentation stage. The gas volume in all groups that added 1%, 2%, and 3% SBP was higher than that of the control group during the 0–2 h, 2–4 h, and 4–6 h periods (*p* < 0.05). At 6–8 h, only the 2% SBP group had a significantly higher gas generation than the control group (*p* < 0.05). No significant differences were observed in the subsequent time periods (*p* > 0.05). It is noteworthy that there was no significant difference in the total gas generation within 48 h among all treatment groups (*p* > 0.05), but the value of the 2% SBP group was the highest, followed by the 1% and 3% SBP groups. Regarding the gas generation parameters, the maximum gas generation potential did not change among the groups, while the gas generation rate showed a linear increase with the increase in SBP level (*p* < 0.05).

As presented in Table 3, the effects of SBP on the nutrient degradability of DM and CP in the feed after in vitro ruminal fermentation are shown. The results showed that with the increase in SBP addition, the digestibility of DM showed a linear increasing trend (*p* < 0.05). The DM digestibility of rumen in vitro fermentation in the 2% and 3% SBP added groups was significantly higher than that in the control group and the 1% SBP added group (*p* < 0.05), with the 2% SBP addition group having the highest DM digestibility. In addition, the CP digestibility of the 2% SBP added group was higher than that of other experimental groups, but there was no significant difference among the groups.

### 3.2. Effects of SBP on Rumen In Vitro Fermentation Parameters of Hu Sheep

The effects of SBP on the pH value, NH3-N, and VFA of rumen in vitro fermentation parameters are shown in Table 4. The results showed that after 48 h of in vitro fermentation, the pH value of the rumen fermentation broth in the group supplemented with SBP was significantly higher than that in the control group (*p* < 0.05). There was no significant difference in pH values between the 2% and 3% SBP addition groups, but it was significantly higher than the 1% addition group (*p* < 0.05). Compared with the control group, the addition of SBP had no significant effect on the NH_3_-N content in the rumen in vitro fermentation broth. There was no significant difference in the total volatile fatty acid and acetic acid content among the experimental groups. The propionic acid content in the 2% and 3% SBP added groups was significantly lower than that in the control group (*p* < 0.05). The ratio of acetic acid to propionic acid in the SBP addition groups was significantly higher than that in the control group (*p* < 0.05). Compared with the control group, the contents of butyric acid, isobutyric acid and valeric acid in the 2% and 3% SBP addition groups were significantly increased (*p* < 0.05).

### 3.3. Effects of SBP on Rumen In Vitro Fermentation Microbial Community Diversity of Hu Sheep

**Figure 1:**
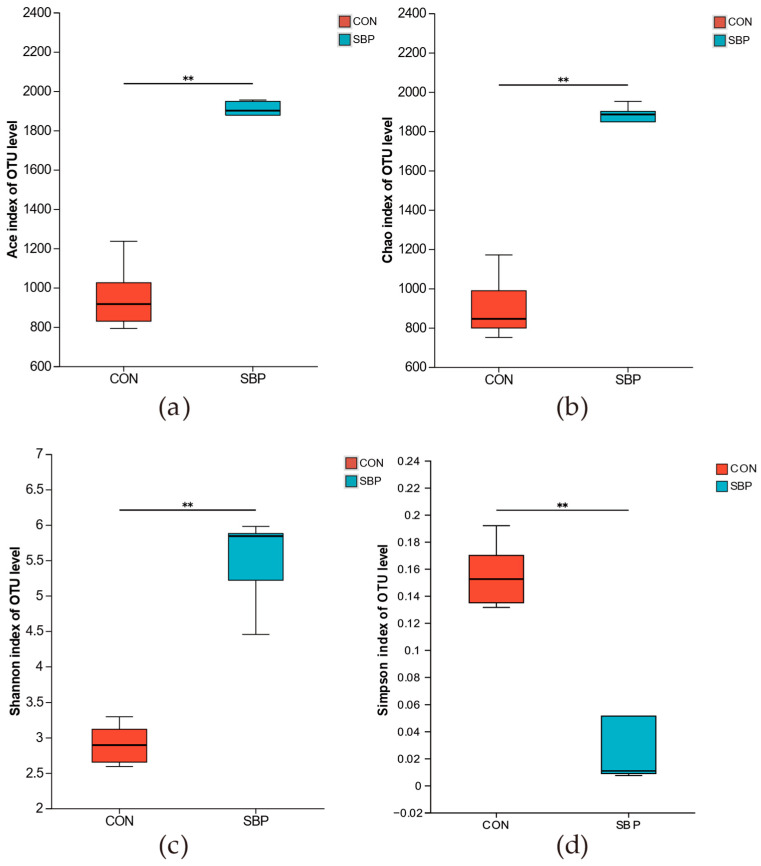
Effects of SBP supplementation on the alpha diversity of the bacterial community in the rumen fluid of Hu sheep. (**a**) Ace index; (**b**) Chao index; (**c**) Shannon index; (**d**) Simpson index. CON = without supplementation SBP; SBP = supplementation with 2% SBP based on DM weight. ** *p* < 0.01.

In this experiment, the control group and the 2% SBP group were selected for 16S rDNA high-throughput sequencing analysis. The effect of SBP on the rumen in vitro fermentation bacterial diversity index of Hu sheep is shown in Figure 1. Compared with the control group, the Ace index, Chao1 index and Shannon index of the rumen in vitro fermentation bacterial flora in the 2% SBP addition group were significantly increased (*p* < 0.05), while the Simpson index was significantly decreased (*p* < 0.05).

**Figure 2:**
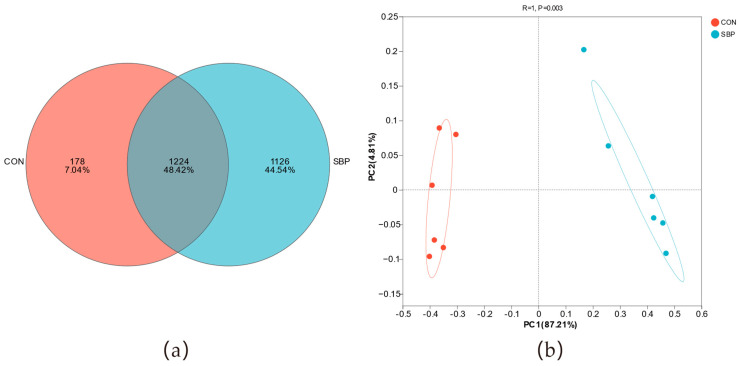
Effects of SBP supplementation on the beta diversity of the bacterial community in the rumen fluid of Hu sheep. (**a**) Venn diagram of microbial community; (**b**) PCoA of the bacterial community.

Based on 97% species similarity, a total of 2528 OTUs were obtained in all samples, including 1224 common OTUs, 1126 unique OTUs in the SBP group, and 178 unique OTUs in the control group (Figure 2a). The rumen in vitro fermentation bacterial community structures of the control group and the 2% SBP treatment group were evaluated by principal coordinate analysis (PCoA), and the results are shown in Figure 2b. In the bacterial principal coordinate analysis, the contribution values of principal coordinate 1 and principal coordinate 2 were 87.21% and 4.81%, respectively. Meanwhile, there was a clear distinction between the rumen in vitro fermentation bacterial flora of the control group and the 2% SBP group, indicating that the addition of 2% SBP had a significant effect on the overall structure of the rumen bacterial microbiota.

### 3.4. Effects of SBP on Rumen In Vitro Fermentation Bacterial Community Composition of Hu Sheep

**Figure 3:**
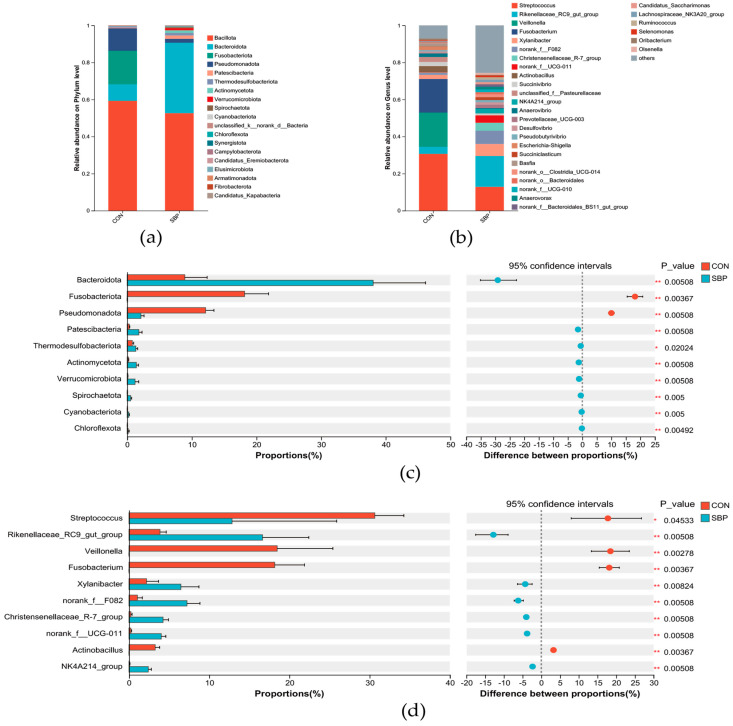
Effects of SBP supplementation on bacteria in the rumen fermentation broth of Hu sheep at the phylum and genus levels. The relative abundances of bacterial (**a**) phyla and (**b**) genera. Wilcoxon rank-sum test bar plot on (**c**) phylum level and (**d**) genera level. * indicates *p* < 0.05; ** indicates *p* < 0.01.

The composition and differences in microbial communities were analyzed at the phylum and genus levels, and the results are shown in Figure 3. At the phylum level, the dominant phyla in the control group and the SBP group were Bacillota and Bacteroidota (Figure 3a). At the genus level (Figure 3b), Streptococcus and Rikenellaceae_RC9_gut_group were the dominant genera. Compared with the control group, the relative abundance of Fusobacteriota and Pseudomonadota in the SBP group was significantly decreased (*p* < 0.05), while the relative abundance of Bacteroidota, Patescibacteria, Thermodesulfobacteriota, Actinomycetota and Verrucomicrobiota was significantly increased (*p* < 0.05) (Figure 3c). The addition of SBP significantly decreased the relative abundance of Streptococcus, Veillonella and Fusobacterium (*p* < 0.05), while significantly increased the relative abundance of Rikenellaceae_RC9_gut_group, Xylanibacter, norank_f__F082, Christensenellaceae_R-7_group, norank_f__UCG-011 and NK4A214_group (*p* < 0.05) (Figure 3d).

### 3.5. KEGG Functional Difference Analysis of Microbial Community

**Figure 4:**
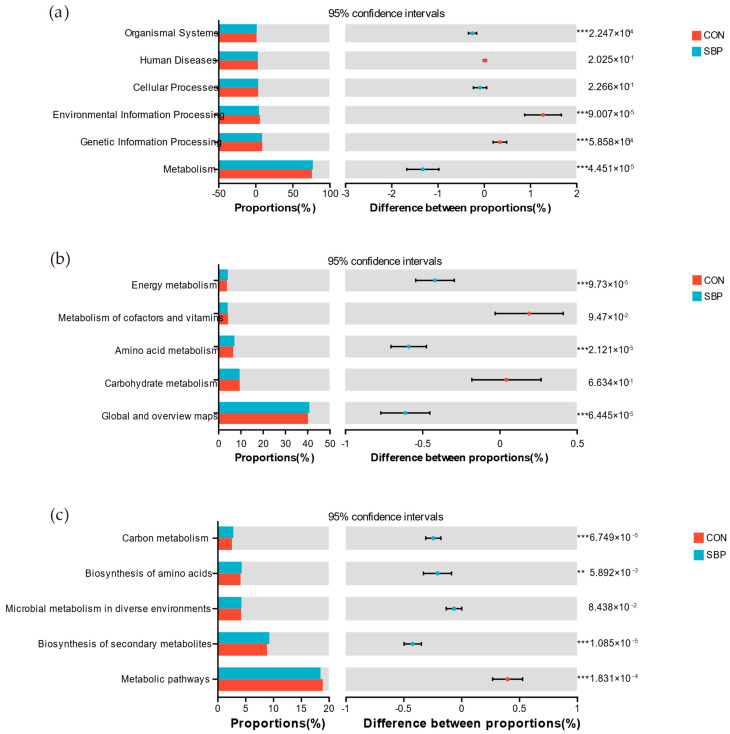
Differential analysis of bacteria in rumen fermentation broth (**a**) at KEGG signaling pathway level 1, (**b**) level 2, and (**c**) level 3. ** Indicates a difference at the statistical significance level of *p* < 0.01. *** indicates a difference at the statistical significance level of *p* < 0.001.

The KEGG pathway classification results showed that in the first-level classification of KEGG pathways, the differential genes of rumen microorganisms were mainly enriched in Organismal Systems, Environmental Information Processing, Genetic Information Processing and Metabolism (Figure 4a). In the second-level classification of KEGG pathways, compared with the control group, the gene expressions of the energy metabolism and amino acid metabolism categories were significantly up-regulated after adding SBP (*p* < 0.05, Figure 4b). In the third-level pathways (Figure 4c), pathways such as carbohydrate metabolism, biosynthesis of amino acid and secondary metabolite biosynthesis were significantly enriched in the SBP group (*p* < 0.05).

## 4. Discussion

### 4.1. Analysis of SBP’s Effects on Rumen In Vitro Fermentation Gas Production and Nutrient Degradability of Hu Sheep

The rumen is the core organ of the ruminant digestive system, acting as a natural and efficient “bioreactor” that harbors complex microbial ecosystems which efficiently ferment feed substrates, facilitating nutrient utilization and energy acquisition for the host [20]. A large number of microorganisms, such as bacteria, fungi, archaea and protozoa, inhabit the rumen, which can convert carbohydrates in the diet into volatile fatty acids to provide energy for the host, and also produce gases mainly including methane, carbon dioxide, and hydrogen [21]. Total gas production is a key indicator reflecting the degree of feed degradation and microbial activity. The higher the degree of degradation in feed, the greater the gas production, indicating a higher activity of microorganisms in the rumen [22,23]. This study found that compared with the control group, the addition of SBP significantly increased the rumen in vitro fermentation gas production at 2~6 h. This suggests that SBP may primarily exert its effects by enhancing initial fermentation kinetics. It is speculated that SBP, as a functional carbohydrate added to feed, provides a rapidly utilizable carbon source for rumen microorganisms, improves the activity of these microorganisms, and enhances the metabolic process of dietary nutrients. This further promotes the rapid decomposition of organic matter and stimulates the production of total fermentation gas. This also indicates that 2% SBP addition has a good effect on promoting rumen fermentation.

The degradability of nutrients is an indicator of the degree of degradation of feed by microorganisms in the rumen, which reflects the efficiency of the breakdown of indigestible nutrients such as plant fibers in the rumen [24,25]. The literature reports that adding artemisia ordosica crude polysaccharides to the diet of cashmere goats can significantly improve the digestibility of feed DM. It is speculated that this effect may be related to polysaccharides improving the rumen microbial flora, promoting the degradation of cellulose in feed, and thus improving the digestibility of nutrients [26]. Guo et al. demonstrated that Jerusalem artichoke polysaccharides (JAP) can regulate rumen microbial communities and metabolites, thereby improving growth and production performance in fattening lambs [5]. This aligns with the hypothesis of SBP’s prebiotic effect and its promotion of microbial growth in this study. Notably, unlike JAP, which primarily targets growth performance and meat quality, SBP more directly enhances rumen in vitro fermentation efficiency by increasing cumulative gas production and DM degradation rate within 48 h. This may be attributed to SBP’s role as a naturally derived bioactive compound with prebiotic properties. It functions both as a microbial carbon source and growth stimulant, thereby optimizing the rumen fermentation environment. This enhances rumen microbial communities and metabolic activity, ultimately influencing nutrient degradation efficiency during in vitro fermentation. SBP is a plant-derived additive that functions both as a microbial carbon source and growth stimulant, thereby optimizing the rumen fermentation environment. This enhances rumen microbial communities and metabolic activity, ultimately influencing nutrient degradation efficiency during in vitro fermentation.

This study also found that with the increase in SBP addition, the DM degradability showed a certain dose dependence, which was similar to the previous research results on astragalus polysaccharides [27]. This indicates that an appropriate amount of SBP can enhance fermentation efficiency by optimizing rumen fermentation characteristics, whereas excessive supplementation may impair degradation effectiveness. The adverse effects observed at high doses can be attributed to increased microbial metabolic load and substrate competition. Such competition may arise from rivalry among carbohydrate types for microbial enzyme resources or from competition among microbial species for energy and nutrients, thereby compromising balanced degradation of the entire diet [28].

### 4.2. Analysis of SBP’s Effects on Rumen In Vitro Fermentation Parameters of Hu Sheep

Rumen fermentation parameters include pH value, NH_3_-N concentration and volatile fatty acid concentration, which comprehensively reflect the fermentation status, environmental changes and animal health in the rumen [29]. PH value is an important indicator for determining rumen homeostasis. This study found that the addition of SBP significantly increased the pH value of rumen in vitro fermentation broth, and the research results were similar to those reported by Liu et al. [30]. The normal range of rumen fermentation pH value in ruminants is 5.5~7.0 [31]. In this study, the pH value of the rumen in vitro fermentation broth was within the normal range, indicating that the addition of SBP would not have a negative impact on the homeostasis of the rumen environment, which is beneficial to the growth and metabolism of microorganisms. NH_3_-N is an important intermediate product of rumen protein degradation, and its concentration reflects the utilization of nitrogen sources by microorganisms and the balance between synthesis and decomposition of rumen protein. Excessively high concentration can lead to increased nitrogen emissions and nitrogen loss, thereby reducing nitrogen use efficiency. Excessively low concentration may inhibit the proliferation of rumen microorganisms and affect the host’s overall utilization of protein [32]. In this study, compared with the control group, the addition of SBP had no significant effect on the content of NH_3_-N, indicating that the addition of SBP had no negative impact on the ammonia utilization and nitrogen metabolism efficiency of rumen microorganisms.

Rumen microorganisms can ferment the crude fiber in the chyme to produce VFAs, which are the main energy source for ruminants. Bioactive polysaccharides and oligosaccharides as possible feed additives to alter rumen fermentation processes and increase the total volatile fatty acid content in Rusitec fermenters [33,34]. In this study, the addition of SBP had no significant effect on the total amount of volatile fatty acids, which was different from previous research results, which may be related to the different polysaccharide structures and addition doses. Consequently, a further detailed analysis of the chemical composition of SBP will be conducted. The combination of lycium barbarum polysaccharides and laminaria japonica polysaccharides as efficient prebiotics can exert complementary advantages to promote the increase in short-chain fatty acids such as butyric acid, isobutyric acid, valeric acid, and isovaleric acid content [35]. This study found that the addition of SBP significantly increased the contents of butyric acid, isobutyric acid and valeric acid, which was similar to previous research results. Butyric acid can be directly used by gastrointestinal epithelial cells to promote gastrointestinal development and regulate systemic energy homeostasis and metabolic function balance [36]. At the same time, butyrate is the main energy source for colon cells, which plays an important role in maintaining intestinal health. Isobutyric acid and valeric acid are branched-chain fatty acids, usually produced by protein fermentation. It is speculated that the increase in their content is related to the indirect promotion of rumen microbial degradation of protein or proliferation of specific microbial groups by SBP. This study provides a theoretical basis for the application of SBP as functional feed additives, but its specific mechanism needs to be further verified through in vivo experiments.

### 4.3. Analysis of SBP’s Effects on Rumen In Vitro Fermentation Microbial Community Diversity of Hu Sheep

Rumen microorganisms play a crucial role in the development of rumen structure and internal fermentation processes, and further affect the growth performance by influencing the digestion and absorption of nutrients [37]. The diversity of rumen microbial communities is affected by factors such as diet composition, feeding methods and feed additives. Li et al. found that adding artemisia annua crude polysaccharides to the diet of cashmere goats increased the Ace index in rumen fluid and decreased the Simpson index [26]. The results of this study showed that the addition of SBP significantly increased the Ace index, Chao1 index and Shannon index of rumen bacteria, and significantly decreased the Simpson index, which was similar to previous research results. This indicates that SBP can enhance the richness, diversity and degree of homogeneity of rumen microorganisms to a certain extent and may optimize the microbial community composition by promoting the growth of certain functional microbial. At the OTU level, the number of unique OTUs in the SBP group was significantly higher than that in the control group, indicating that SBP may promote the proliferation of specific bacterial groups. PCoA further showed that there was a clear separation in bacterial community structure between the control group and the SBP treatment group, indicating that SBP is the main factor driving variations in microbial community structure. This result is consistent with the changes in diversity index, indicating that SBP have a significant regulatory effect on the structure and composition of in vitro fermentation bacteria in the rumen of Hu sheep.

### 4.4. Analysis of SBP’s Effects on Rumen In Vitro Fermentation Bacterial Community Composition and Its Function of Hu Sheep

Bacillota and Bacteroidota are the two dominant phyla in the rumen fluid of ruminants [38]. Bacteroidota is mainly responsible for decomposing proteins and non-fibrous carbohydrates in feed, while Bacillota is mainly responsible for decomposing fibrous carbohydrates in the rumen [23]. VFA produced by the decomposition of non-fibrous carbohydrates and fibrous carbohydrates provide an important energy source for ruminants. The results of this study showed that compared with the control group, the addition of SBP significantly increased the relative abundance of Bacteroidota, which was consistent with the study that adding tea polysaccharides to the diet of beef cattle increased the relative abundance of Bacteroidetes in the rumen [39], indicating that SBP can promote the growth and reproduction of Bacteroidota microorganisms and change the relative abundance of dominant bacterial groups. Actinomycetota contains probiotics such as *Bifidobacterium*, which can protect the host from intestinal pathogenic infections by producing acetate [40]. Verrucomicrobiota is rich in genes related to lignocellulose polymer decomposition and can improve the degradation of cellulose into available VFAs [41,42]. This study found that the addition of SBP significantly increased the relative abundance of Actinomycetota and Verrucomicrobiota, which was consistent with previous research results [43]. It is speculated that the significant increase in the relative abundance of these two bacteria promotes the conversion of feed nutrients and provides beneficial effects on the gastrointestinal health of Hu sheep. In addition, the addition of SBP significantly increased the relative abundance of Patescibacteria and Thermodesulfobacteriota, indicating that SBP may provide available carbon or energy sources for these two bacterial phyla, promoting their growth and metabolic activities [44].

At the genus level, we observed that the addition of SBP significantly reduced the relative abundance of bacterial genera usually associated with pro-inflammatory or pathogenic states, including *Streptococcus*, *Veillonella* and *Fusobacterium*. It is speculated that the inhibition of these genera may help reduce inflammation in the intestinal environment. *Christensenellaceae_R-7_group* is a member of Christensenellaceae. According to literature reports, Christensenellaceae is positively correlated with the proteolytic metabolism of animal protein in feed. In addition, *Christensenellaceae_R-7_group* can promote the development of the rumen of ruminants and increase the digestion and absorption of nutrients [45]. This study found that the addition of SBP significantly increased the relative abundance of *Christensenellaceae_R-7_group*, and the nutrient degradability of feed DM and protein increased significantly, which also confirms the conclusions of previous research. The *norank_f__F082* genus belongs to the phylum Bacteroidetes, which is mainly responsible for decomposing non-cellulosic substances, and its metabolites are mainly propionic acid and butyric acid [46]. In this study, it was also found that the content of butyric acid in the rumen fluid increased significantly, which is speculated to be related to the significant increase in the relative abundance of this bacterium caused by the addition of SBP. Studies have found that *norank_f__UCG-011* and *NK4A214_group* are positively correlated with acetate and acetate/propionate ratio in the rumen of Chinese Holstein cows [47]. The results of this study showed that the addition of SBP significantly increased the acetate/propionate ratio and the relative abundance of this genus in the rumen of Hu sheep, which was similar to the results of this study. In addition, the addition of SBP also significantly increased the enrichment of beneficial bacteria *Xylanibacter* and *Rikenellaceae_RC9_gut_group* related to fiber degradation and intestinal health maintenance. Previous studies have found that Paeonia lactiflora polysaccharides can slow down the decrease in beneficial intestinal bacteria *Rikenellaceae_RC9_gut_group* caused by LPS exposure [48], which is similar to the results of this study.

Through the prediction of rumen microbial gene function, it was found that the differentially enriched KEGG pathways were mainly related to energy metabolism and amino acid metabolism. KEGG functional genes related to carbohydrate metabolism and amino acid biosynthesis were enriched in the SBP group, indicating that dietary supplementation with SBP altered both the structure of the rumen microbial community and its metabolic potential. This enhanced carbohydrate and protein metabolic capacity, promoting the conversion of substrates into short-chain fatty acids and amino acids. This further confirms the impact of SBP on rumen microbial composition.

## 5. Conclusions

This study indicates that adding 2% SBP to the diet significantly increased the relative abundance of beneficial bacterial, including *Rikenellaceae_RC9_gut_group*, *Xylanibacter*, *norank_f__F082*, *Christensennellacee_R-7_group*, *norank_f__UCG-011*, and *NK4A214_group*, in the rumen in vitro fermentation broth, while the relative abundance of opportunistic pathogens, such as *Streptococcus*, *Veillonella*, and *Fusobacterium*, significantly decreased. Moreover, DM degradability, 48 h cumulative gas production, and the content of acetate/propionate, butyric acid, isobutyric acid, and valeric acid in the rumen fermentation broth were significantly increased. This phenomenon may be related to the increased diversity and compositional changes in rumen microbial communities. In addition, the functions of rumen microbiota in the SBP group showed significant enrichment in carbohydrate metabolism, biosynthesis of amino acid, and secondary metabolite biosynthesis. In summary, this study showed that the optimal feeding amount of SBP for rumen fermentation in Hu sheep is 2%, but its in vivo effects need further validation.

## Figures and Tables

**Table 1:** Composition and Nutrient Levels of Total Mixed Ration for Experimental Sheep (air dry basis).

Items	Content (%)	Nutrient Levels	Content (%)
Corn	29.8	DE (MJ/Kg)	11.48
Corn stover	23	CP	18.34
Alfalfa hay	6	EE	2.12
Cottonseed meal	13	NDF	33.32
Distillers dried grains with solubles (DDGS)	5	ADF	10.76
Spray-dried corn husk	5	Ca	0.71
Chili skin residue	6	P	0.42
Wheat middlings	9		
Limestone powder	1.4		
Sodium bicarbonate	0.8		
Salt	0.5		
Premix	0.5		
Total	100		

Note: DE, Digestible energy; CP, Crude protein; EE, Crude fat; NDF, neutral detergent fiber; ADF, acid detergent fiber. The premix provides per kilogram of diet: VA 1,200,000 IU, VD 500,000 IU, VE 5000 IU, Fe 8 g, Zn 10 g, Cu 1.6 g, I 60 mg, Mn 6 g, Se 60 mg, Co 60 mg. Digestible energy is a calculated value, and others are measured values.

**Table 2:** Effects of SBP on Rumen In Vitro Fermentation Gas Production of Hu Sheep.

Item	SBP Levels				SEM	*p*-Value		
	CON	1%	2%	3%		ANOVA	Linear	Quadratic
Gas production (mL/g DM)								
0~2 h	2.65 ^b^	7.24 ^a^	8.28 ^a^	9.62 ^a^	0.77	0.003	0.000	0.181
2~4 h	2.64 ^b^	7.24 ^a^	8.94 ^a^	9.63 ^a^	0.81	0.003	0.001	0.132
4~6 h	3.31 ^b^	7.90 ^a^	10.27 ^a^	10.61 ^a^	0.91	0.007	0.001	0.156
6~8 h	7.61 ^b^	9.88 ^ab^	13.58 ^a^	12.60 ^ab^	0.91	0.075	0.020	0.339
8~10 h	6.94	12.18	13.25	10.28	1.06	0.162	0.232	0.054
10~12 h	7.94 ^ab^	6.92 ^ab^	9.27 ^a^	5.31 ^b^	0.58	0.090	0.257	0.181
12~24 h	32.41	43.11	40.08	46.77	3.16	0.448	0.174	0.756
24~36 h	19.51	26.66	22.85	18.90	1.78	0.410	0.726	0.134
36~48 h	18.86	18.11	22.84	13.93	1.53	0.241	0.458	0.184
Total gas	101.87 ^b^	138.18 ^ab^	149.35 ^a^	137.64 ^ab^	7.05	0.081	0.052	0.076
Gas production parameters								
Maximum gas production/mL	107.56	139.40	146.04	137.74	6.83	0.195	0.112	0.140
Gas production ratec/(%/h)	0.08	0.08	0.11	0.10	0.00	0.129	0.038	0.888

Note: ^a,b^ means in the same row with different superscripts are significantly different (*p* ≤ 0.05).

**Table 3:** Effects of SBP on Nutrient Digestibility of Rumen In Vitro Fermentation of Hu Sheep.

Item	SBP Levels				SEM	*p*-Value		
	CON	1%	2%	3%		ANOVA	Linear	Quadratic
IVDMD, %	53.56 ^b^	54.16 ^b^	59.61 ^a^	57.81 ^a^	0.73	0.002	0.001	0.283
IVCPD, %	42.46	42.46	47.75	42.27	1.14	0.256	0.640	0.231

Note: ^a,b^ means in the same row with different superscripts are significantly different (*p* ≤ 0.05); IVDMD: in vitro DM digestibility; IVCPD: in vitro Crude Protein digestibility.

**Table 4:** Effects of SBP on Rumen In Vitro Fermentation Parameters of Hu Sheep.

Item	SBP Levels				SEM	*p*-Value		
	CON	1%	2%	3%		ANOVA	Linear	Quadratic
pH	6.41 ^c^	6.79 ^b^	6.91 ^a^	6.92 ^a^	0.05	0.000	0.000	0.000
NH_3_-N (mg/dL)	10.28	10.76	11.43	10.62	0.23	0.372	0.416	0.177
Total VFA (mM)	173.50	167.04	169.52	177.58	2.10	0.330	0.242	0.861
Concentration (mol/100 mol)								
Acetate	115.53	114.76	114.92	120.40	1.37	0.423	0.493	0.730
Propionate	48.02 ^a^	40.38 ^b^	41.50 ^b^	43.39 ^ab^	0.99	0.022	0.026	0.308
Acetate /Propionate	2.41 ^b^	2.88 ^a^	2.77 ^a^	2.78 ^a^	0.05	0.001	0.004	0.038
Butyrate	8.58 ^c^	10.52 ^b^	11.55 ^ab^	12.23 ^a^	0.36	0.000	0.334	0.771
Isobutyrate	0.38 ^b^	0.39 ^b^	0.42 ^a^	0.43 ^a^	0.01	0.003	0.557	0.286
Valerate	0.39 ^b^	0.42 ^b^	0.51 ^a^	0.53 ^a^	0.02	0.002	0.419	0.214
Isovalerate	0.60	0.56	0.61	0.62	0.01	0.205	0.176	0.142

Note: ^a,b^ means in the same row with different superscripts are significantly different (*p* ≤ 0.05).

## Data Availability

The original contributions presented in this study are included in the article. Further inquiries can be directed to the corresponding author.
